# Kidney supportive care: an update of the current state of the art of palliative care in CKD patients

**DOI:** 10.1590/2175-8239-JBN-2020-0017

**Published:** 2020-09-04

**Authors:** Alze Pereira dos Santos Tavares, Cássia Gomes da Silveira Santos, Carmen Tzanno-Martins, José Barros, Ana Maria Misael da Silva, Leda Lotaif, Jonathan Vinicius Lourenço Souza

**Affiliations:** 1 Sociedade Brasileira de Nefrologia, Comitê de Cuidados Paliativos, São Paulo, SP, Brasil.; 2 Hospital Santa Paula, São Paulo, SP, Brasil.; 3 Universidade Federal do Paraná, Hospital das Clínicas, Curitiba, PR, Brasil.; 4 Clínica de Hemodiálise, São Paulo, SP, Brasil.; 5 Sociedade Brasileira de Nefrologia Mineira, Belo Horizonte, MG, Brasil.; 6 Felício Rocho Hospital, Departamento de Nefrologia, Belo Horizonte, MG, Brasil.; 7 Instituto Dante Pazzanese de Cardiologia, Nefrologia e Hipertensão e Pós-Graduação, São Paulo, SP, Brasil.; 8 HCor, São Paulo, SP, Brasil.

**Keywords:** Renal Insufficiency, Chronic, Palliative Care, Conservative Treatment, Insuficiência Renal Crônica, Cuidado Paliativo, Tratamento Conservador

## Abstract

Chronic kidney disease (CKD) has become a public health burden worldwide for its increasing incidence and prevalence, high impact on the health related quality of life (HRQoL) and life expectancy, and high personal and social cost. Patients with advanced CKD, in dialysis or not, suffer a burden from symptoms very similar to other chronic diseases and have a life span not superior to many malignancies. Accordingly, in recent years, renal palliative care has been recommended to be integrated in the traditional care delivered to this population. This research provides an updated overview on renal palliative care from the relevant literature.

## RESUMO

A doença renal crônica (DRC) tornou-se um peso na saúde pública em todo o mundo por sua crescente incidência e prevalência, seu alto impacto na qualidade de vida relacionada à saúde (QVRS) e na expectativa de vida, e alto custo pessoal e social. Pacientes com DRC avançada, em diálise ou não, sofrem de uma carga de sintomas muito semelhantes aos de outras doenças crônicas, e têm uma sobrevida não superior àquela de muitas doenças malignas. Assim, nos últimos anos, recomenda-se que os cuidados paliativos renais sejam integrados aos cuidados tradicionais prestados a essa população. Este trabalho fornece uma visão geral atualizada sobre os cuidados paliativos renais discutidos na literatura relevante.

## Introduction

The history of renal palliative care dates back to the early 1980s when American nephrologists began discussing the practice of dialysis withdrawal in fragile patients with serious comorbidities^1^^,^ 2. However, it was from the publication of the Clinical Practice Guideline on Shared Decision-Making in the Appropriate Initiation of and Withdrawal from Dialysis^3^, later updated in 2010^4^, that renal palliative care was developed in a more structured way, mainly in countries such as Australia, Canada, and the United Kingdom. The concept of conservative management (without dialysis) of end-stage renal failure, also called “comprehensive conservative care (CCC)^5^” and “conservative kidney management (CKM)^6^” was introduced in some renal units in the UK since 2003^7^^,^ 8 and is currently a treatment option established in most nephrology services in the UK and other countries. Finally, in 2015, an executive summary with a roadmap to best practices in renal supportive care under the KDIGO seal is published for the first time^5^.

The World Health Organization defines Palliative Care as an approach that improves the quality of life of patients (adults and children) and their families who are facing problems associated with life-threatening illness. It prevents and relieves suffering through the early identification, correct assessment, and treatment of pain and other problems, whether physical, psychosocial, or spiritual^9^.

Renal palliative care (RPC) is an interdisciplinary model of person-centered medicine that seeks to optimize health-related quality of life (HRQoL) and preserve human dignity through strategies such as adequate communication with patient and family, shared decision making, planning future health care/treatment, and management of pain and other biopsychosocial and spiritual problems, including grief and proper end-of-life care^5^.

## Discussion

### Epidemiological and Clinical Features

Patients with advanced chronic kidney disease (CKD) present a high burden of stressful physical and psychological symptoms^10^^-^ 12, similar to what occurs in other chronic diseases, such as cancer^13^^,^ 14. This cluster of symptoms has a negative impact on quality of life, and symptoms evaluation, despite progresses done in the last decades, is still overlooked by many nephrologists^15^. In addition, incidence and prevalence of dialysis in patients over 75 years of age have increased and are the fastest growing palliative population in recent years^16^^-^ 18.

Although dialysis and renal transplantation significantly increase life expectancy and allows a reasonable quality of life in selected elderly with renal impairment, most of these patients present with severe comorbidities or geriatric syndromes such as frailty, functional disability, or dementia that tend to worsen with the onset of dialysis^19^^-^ 23. The annual mortality rate of patients on dialysis is about 20-25% in the general population and approximately 38% for those aged 75 years or older^17^, but in fragile elderly patients it may exceed 50%^23^. Data from United States Renal Data System (USRDS) indicate that dialysis withdrawal precedes death in about a quarter of patients with end-stage renal disease (ESRD)^16^, a possible reflection of low HRQoL in this population. Furthermore, the most common cause of dialysis death in Australia appears to be withdrawal related to psychosocial or progressive chronic diseases^24^. Whilst in the UK, death from withdrawing remains the fourth highest cause of death in patients of all ages undergoing chronic dialysis, after cardiovascular diseases, infection, and other causes^25^. In addition, current evidence suggests that end-of-life care practices are not consistent with the preferences of patients with advanced CKD^23^. Most patients with CKD want to be fully informed about their disease (80.6%) and prognosis (78.3%)^26^. Besides, ~19% regretted to start dialysis and 41% preferred comfort care rather than prolonging life^26^.

Although many older people who initiate dialysis are likely to live longer than those receiving comprehensive conservative care (CCC), this advantage may be small or non-existent in patients with severe comorbidities, particularly cardiovascular disease, dementia, and diabetes^27^^-^ 29. In a cohort of older patients with ESRD, Verberne et al. found that patients aged ≥70 years choosing dialysis had better survival compared with patients choosing CCC^30^. However, this survival advantage was lost in patients aged ≥ 80 years. They also observed a considerable negative effect of comorbidity on survival, particularly of cardiovascular comorbidity. These results indicate that CCC could be a valid treatment option in selected patients^30^. In addition, the dialysis burden and its effect on quality of life may outweigh the benefit of longevity for some renal patients^31^^-^ 34. In a discrete-choice experiment (DCE) involving stage 3-5 CKD, patients were willing to give up 7 and 15 months of life expectancy to reduce the number of visits to the hospital or increase their ability to travel, respectively^35^. In another DCE, Australian nephrologists were willing to abandon 12 months of patient survival to avoid a substantial decrease in HRQoL related to dialysis^36^. On the other hand, the important role of CCC as an alternative to dialysis in patients with advanced CKD who refuse dialysis and in elderly over 75 years old who present with severe comorbidities, frailty or dementia is increasingly recognized^37^^-^ 40. Currently, in high income countries, up to 15% of patients with advanced CKD, for various reasons, choose not to dialyze and are maintained in CCC^41^.

### Diagnosis and Management of CKD Under A Palliative Care Perspective

Palliative Care is a specialized and transdisciplinary approach^9^ that has emerged in response to clear inadequacies in the management of patients with severe and complex diseases. It is applied in any age group and is not incompatible with curative, stabilizing, or disease modifying treatments. In recent years there has been increasing recognition that palliative care principles applied earlier in the disease trajectory, according to patients’ needs, improve outcomes and patient experience and even positively influence survival^42^.

**Figure 1:**
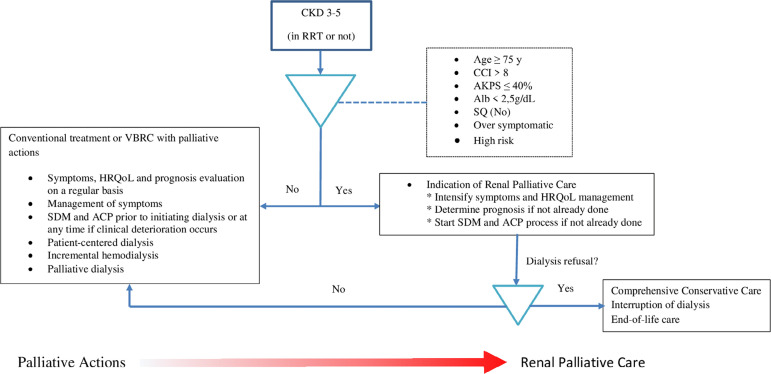
Flowchart: renal healthcare unit with an integrated renal palliative care service.

Abbreviations: CKD, chronic kidney disease; RRT, renal replacement therapy; HRQoL, health related quality of life; SDM, shared-decision making; ACP, advance care planning; CCI, Charlson comorbidity index; AKPS, Australian-modified Karnofsky performance scale; Alb, albumin; SQ, the surprise question, ‘‘Would you be surprised if this patient died within the next six months?’’; VBRC, value-based renal care. Adapted from Scherer JS et al. (ref. 43).

At first, every patient with CKD would have, to a lesser or greater degree, an indication of palliative care, especially those who are in the more advanced stages of the disease, on dialysis or not (Figure 1). Therefore, in order to diagnose the palliative care needs of a CKD patient at any stage, we must explore and implement stablished strategies of palliative medicine.

It is paramount that the beneficial integration of the strategies and actions of palliative medicine (Table 1) begin early and continue along the trajectory of the renal disease^40^^,^ 41.

**Table 1:** Kidney palliative care strategies and actions

Strategy	Action
Management of symptoms and quality of life	Evaluate symptoms on a regular basis with the use of validated tools and adopt effective measures for control
Prognostic	Evaluate prognosis on a regular basis with the use of validated tools
Communication	Establish a patient-centered communication and explore patient values and preferences
Shared decision making	Promote shared decision-making on renal replacement therapy options, including dialysis and non-dialytic control of chronic kidney disease
Advance care planning	Explore patient values and preferences in advance about unwanted health care when they lose the ability to make decisions for themselves and realistic life goals they would like to achieve. Advance directives of will are part of this process
Comprehensive conservative care	Evaluate, select, and provide comprehensive conservative care to individuals who are unlikely to benefit from dialysis
Patient-centered dialysis	Personalize treatment and deliver it with dignity, compassion, and respect to the patient values and preferences
Incremental hemodialysis	Implement incremental HD according to the metrics of residual kidney function to reduce the burden of treatment
Palliative dialysis	Implement palliative or “comfort” dialysis and tailor it to individual patient needs to optimize quality of life and minimize burden of treatment
Dialysis withdrawal	Develop written guidelines on how and when to discuss dialysis withdrawal and how to manage patients after withdrawal
End-of-life care	Explore patient preferences for end-of-life care when life expectancy is less than 12 months

HD, hemodialysis

#### 1. Management of symptoms and quality of life

Patient-reported outcome measures (PROMs) and patient-reported experience measures (PREMs) are now considered the gold standard in assessing the quality of health services provided to the population and as a consequence a paramount component for improvement of the healthcare system. Evaluation of symptoms in patients with CKD should be done at regular intervals and preferably with tools validated for this population^43^^-^ 46. Considering that CKD patients have a mean of 6 to 20 simultaneous symptoms and that they may have important interactions (such as pruritus and insomnia)^43^^-^ 48, the use of tools that evaluate multiple symptoms are the most recommended. Ideally, these tools should be multidimensional and evaluate characteristics such as the prevalence, intensity, frequency, and impact of each symptom on the quality of life and have a recall period of up to one week^44^. Regarding the instruments used to assess quality of life, given its greater complexity, their application is usually performed at intervals ranging from 3 months to one year. Table 2 presents some tools currently used for this purpose.

**Table 2:** Patient-reported outcome measures (PROMs) in CKD

Instrument	Population	Number of symptoms	Time to complete	Recall period	Frequency of symptom	Impact of symptom on HRQoL
ESAS-r: Renal	Dialysis	10	NR	Present	N	N
IPOS-Renal	Non-dialysis	15	< 10 min	1 week	N	N
DSI	Dialysis	30	NR	1 week	N	N
CKD-SBI	Dialysis and Non-dialysis	32	NR	4 weeks	Y	Y
KDQOL-36*	Dialysis and Non-dialysis	30	> 30 min	4 weeks	Y	Y**

* Instrument used to evaluate HRQoL;

** Only for pain, sexual dysfunction, sleep and fatigue. Abbreviations: ESAS-r: Renal, Edmonton symptom assessment system-revised: Renal; IPOS-Renal, integrated palliative care outcome scale-renal; DSI, dialysis symptom index; CKD-SBI, chronic kidney disease-symptom burden index; KDQOL, kidney disease quality of life instrument; HRQoL, health-related quality of life; Y, yes; N, no; NR, not reported.

The evaluation of symptoms should occur at regular intervals and according to the recall period of the chosen tool (Table 2). The interval can be equal to or greater than the recall period, but never shorter. The IPOS-Renal, with a one-week recall,^49^ and ESAS-r: Renal, which evaluates the present symptoms^44^, are recommended tools for routine screening at each consultation^50^. Recently, the International Consortium for Health Outcomes Measurement (ICHOM), in the CKD standard set for value-based health care (VBHC), recommended PROMs use like PROMIS-Global Health or SF-36v2 every 6 months^51^^,^ 52. Table 3 summarizes the pharmacological treatment of the most common symptoms in CKD.

**Table 3:** Evidence-based symptom management in CKD

Symptom	Medication	Comments
Pain	1. Mild pain (1-3) - Dipyrone or Paracetamol (max. of 3g daily) 2. Moderate pain (4-6) - Tramadol with reduced dose. On dialysis 50-100mg 2x / d (maximum dose). In conservative 5-50mg 2x / d (maximum dose). Some authors recommend skip step 2 in CKD. 3. Severe Pain (7-10) - Fentanyl, Buprenorphine, Hydromorphone and Methadone are considered safe. Start with small doses.	Assess the cause of pain. Reduction of 20-30% in pain intensity is sufficient to improve HRQoL. Administer analgesic medication according to WHO principles: by mouth, by the clock, by the ladder, for the individual and with attention to detail. Neuropathic or mixed pain requires another class of medications as SSRI, TCAs, and Gabapentinoids.
Uremic pruritus	Gabapentin CKD stage 3 and 4 – start with 50-100 mg 1 – 2 h before sleep CKD stage 5 – start with 100mg on alternate nights Dialysis – start with 100mg after each session and holder for efficacy and side effects	Remove other causes of pruritus. Moisturizing is advisable.
Restless legs syndrome	Gabapentin - as above Dopaminergic agonist - ropinirole 0.5mg at night or pramipexole 0.25mg at night If the patient presents with uremic pruritus and cramp prefer gabapentin	If the patient has uremic pruritus and cramps, prefer gabapentin.
Nausea and vomiting	First line: ondansetron 4 – 8 mg every 8 h as needed. Second line: Metoclopramide 2.5 every 8 h as needed and before meals. Third line: olanzapine 2.5 mg every 8 h as needed or haloperidol 0.5 mg every 8 h as needed.	Multifactorial in origin. Metoclopramide acts as a central and peripheral antiemetic (uremic and diabetic gastroparesis).
Constipation	Bisacodyl or Senna	Add fiber to diet. Check for medications that cause constipation.
Dyspnea	Hydromorphone - start at 0.5mg 4x / d and increase if tolerated Morphine 2.5mg 4x / day for 2 to 3 days	Exclude reversible causes
Anorexia	Remove precipitants Diet review Supplements	Multifactorial
Fatigue	Treat the reversible causes	Multifactorial
Anxiety	Counseling Psychologist / Psychiatrist evaluation If panic attack consider Benzodiazepines - Lorazepam 0.5mg to 1mg.	Multifactorial
Depression	Some SSRIs as Citalopram, Fluoxetine and Sertraline are safe for use in CKD	Difficult to diagnose because the symptoms of depression seem those of the DRCT. Consider Psychiatrist evaluation.
Sleep disturbance	Assess the cause carefully Treat the cause Temazepam 10-20 mg at night	If sleep apnea is suspected - polysomnography

SSRIs, selective serotonin reuptake inhibitors. Adapted from Davison SN et al (ref. 50)

#### 2. Prognostication

Estimating the prognosis of a patient with CKD is of great importance, and the estimation should comply with several purposes such as resource planning, development of a care plan, informed decision making by the patient, and identification of high risk patients who may benefit from an intervention^3^. In addition, several studies have shown that most patients want to know the prognosis and trajectory of their disease^53^. Furthermore, inadequate information with overly optimistic estimates can trigger unrealistic expectations, frustration, anxiety, depression, and inappropriate aggressive treatments^54^. In addition to a respectful communication about their disease and disease progression, patients want physicians to be realistic, patient, trustworthy, and tactful, understand psychosocial needs, provide time for questioning, and individualize their prognosis^55^. Studies with patients in other chronic diseases show that patients are more likely to receive end-of-life care consistent with their preferences when given the opportunity to discuss their wishes for care with a physician^56^^,^ 57.

Appropriate counseling of patients with advanced CKD regarding treatment options depends on a reliable estimate of life expectancy at a given time, with or without dialysis^58^. Studies show that physicians are imprecise in their prognosis about the termination of life and that the error is systematically optimistic^59^. For this reason, the use of prognostic tools for CKD is recommended. Some of these instruments are presented in Table 4.

**Table 4:** Instruments used to assess risk of death in CKD

Tool	Feature
1. The Surprise Question (SQ): "Would you be surprised if this patient died in the next 12 months?"	A simple and useful tool to identify patients at greater risk of death. Applicable at any stage of CKD.^60^
2. AKPS (Australia-modified Karnofsky Performance Status scale) or KPS (Karnofsky Performance Scale)	Tools used to assess the patient's functional status. A patient with AKPS or KPS of less than 50% is eligible for palliative care. That is, a patient who, due to one or more chronic diseases, spends at least 50% of the time sitting or lying down, without considering sleep time.^61^
3. Modified Charlson score (MCS)	A tool that adds comorbidities scores (9 in total) to age scores (an additional point for each decade above the age of 40) to identify patients on dialysis with a higher risk of death.^62^
4. Fried Phenotype Model	Frailty is highly prevalent in CKD and associated with increased risk of hospitalization and mortality.^63^
5. Bansal Score	This tool uses nine clinical variables (age, sex, race, eGFR, albumin / creatinine ratio, diabetes, smoking, heart failure and stroke) to estimate the risk of death at 5 years in the elderly (≥ 70 years) with non-dialytic DRC (stages 3 and 4).^64^
6. REIN Score	REIN Score Instrument validated to stratify the risk of death in 3 months of elderly patients (> 75 years) who intend to initiate dialysis^65^
7. Cohen 6-month Mortality Predictor (available at: http://touchcalc.com/calculators/sq)	An instrument that combines SQ with four variables - age, serum albumin, presence of dementia and peripheral vascular disease - to predict the risk of death at 6, 12, or 18 months for hemodialysis patients.^66^
8. KDIGO Clinical Outcomes in CKD (available at: https://kdigo.org/equation/)	This tool estimates concomitantly the risks of death, RRT, and any cardiovascular disease in 2 and 4 years in patients with CKD with eGFR between 15 and 30 mL/min/1.73m2 and aged between 30 and 85 years.

RRT, renal replacement therapy; eGFR, estimated glomerular filtration rate; REIN, renal epidemiology and information network.

As only a minority of elderly people with CKD will progress to ESRD^67^, it is important to identify those with a higher risk of progression. For this aim it is recommended to use the risk equation for renal failure of Tangri (KFRE of 4 variables)^68^. This instrument uses routine demographic and laboratory variables to predict which patients with CKD stages 3 to 5 will progress to dialysis. Patients at higher risk of progression and at high risk of mortality (with or without dialysis) are eligible for comprehensive conservative care and palliative care.

Other features such as global geriatric assessment, nutritional status assessment, cognitive dysfunction and frailty are considered to be very important and used as prognostic markers for patients with advanced CKD. These tools help in the implementation of preventive, regenerative, and supportive measures in addition to identifying the patients with higher risk of death^69^. Neither of these tools is sensitive or specific enough to allow accurate prediction, so when discussing the future with a patient, a degree of uncertainty must be explicitly mentioned. This allows the doctor to frame the conversation in a way that it is recognized that things may not go as planned, either in the best or worst scenario^70^. Patients with very poor prognosis should be informed that dialysis may not confer a survival advantage, improve quality of life or functional status in relation to CCC^5^.

#### 3. Communication

Nephrologists often face difficult conversations that generate anxiety and insecurity such as giving bad news, discussing prognosis, talking about onset, retention of or withdrawal from dialysis, or about end-of-life care. On the other hand, every person has a unique and individual perception of what HRQoL means to them, that might be not as judged by another person, and this gives all patients and families the right to make informed decisions about treatments^71^. Therefore, ability to communicate with patients and their family is an indispensable skill for the proper practice of medicine. It is considered that the professional has good communication skills when he is able to give information in a clear (understandable) and sensitive way, encourage patient participation, evaluate comprehension effectively, explore values and preferences of care, and respond appropriately to the patients’ emotions^71^. An informed patient is one who, after hearing the news, can repeat the information given, demonstrating understanding. It is known that good communication improves the patient’s experience, adjustment to illness, and adherence to medical treatment^72^^,^ 73.

Communication tools like “Ask-Tell-Ask” encourage two-way communication between doctor and patient, and they should be used to initiate difficult conversations. It is recommended to use open questions and not to give more than three new pieces of information at a time. When patients react to bad news with strong emotion their ability to process any subsequent information is impaired^74^^,^ 75. Therefore, it is important for the nephrologist to recognize and respond to patients’ emotions in a verbal manner (name and understand the emotion, respect and support the patient, and explore the emotion) and non-verbal manner (eye contact, change of position, touch, allow silence)^76^.

#### 4. Shared Decision Making

Shared decision making (SDM) is a communication process whereby physicians and patients agree on a specific course of action based on a common understanding of treatment goals, taking into account the benefits and harms of treatment options and the likelihood of achieving the results that are most important for individual patients. SDM is particularly important before initiating dialysis where patients can understand the benefits, risks, and alternatives to dialysis^5^.

#### 5. Advance care planning

Advance care planning (ACP) is a process involving understanding, communication, and discussion between a patient, family (or other caregiver), and health care staff to clarify preferences for end-of-life care. It establishes a set of relationships, values, and processes to address end-of-life decisions for individuals, including attention to ethical, psychosocial, and spiritual issues related to initiating, continuing, withholding, and discontinuing dialysis^5^. Advance directives (living will, non-resuscitating order, appointment of a decision maker) are part of this process.

#### 6. Comprehensive conservative care (CCC)

Also called conservative management^30^^,^ 77, maximal conservative management^32^, or conservative kidney management^78^, it is a planned patient-centered care for patients with stage 5 CKD. It is indicated for patients unlikely to benefit from dialysis (apply prognostic tools described above) or who choose not to dialyze^5^. In a systematic review of 12 cohort studies, patients choosing dialysis and those opting for conservative management had a median survival time of 8-67 months and 6-30 months, respectively, and median survival is 13 months shorter for CCC patients than dialysis patients 27 ^,^ 77. Literature data are still scarce and controversial, but existing evidence suggests that the survival advantage of dialysis disappears in 75-year-old patients with high levels of comorbidities and/or poor functional status^30^^-^ 34. Recent work has suggested that when asked to choose between dialysis and conservative management, patients are willing to accept a significantly reduced life expectancy in order to reduce the burden and restrictions placed on them by dialysis^35^. In a prospective observational study, authors showed that satisfaction with life did not change overtime in patients in conservative management. However, satisfaction with life decreased significantly after dialysis initiation and did not recover 33. For patients on another prospective conservative care pathway supported by a palliative care team, symptom burden and HRQoL was maintained or improved subsequently in over two thirds of patients^47^. ^( )^In all studies on this topic, patients opting for CCC are older, have high rate of comorbidity and are more dependent than those that embark on dialysis. For ethical and technical reasons, randomized, controlled trials in this area may not be possible for a while. Comprehensive conservative care does not include dialysis. However, the patient may change his/her mind and embark on a dialysis program if he/she wishes. Actions of CCC are described in Table 5.

**Table 5:** Comprehensive Conservative Care

1. Interventions to delay the progression of renal disease and minimize the risk of adverse events or complications
2. Active management of symptoms
3. Shared decision making
4. Detailed communication, including advance care planning
5. Psychological, cultural, and spiritual support
6. Social and family support

Adapted from Davison SN et al. (ref. 5).

#### 7. Patient-Centered Dialysis

There is a growing interest in patient-centered care, defined by the Institute of Medicine as “care that is respectful of and responsive to individual patient preferences, needs, and values”. Although generally accepted as uncontroversial, the notion of “centering” care on our patients is in fact quite revolutionary. Because medical teaching, research, and practice have traditionally been organized around diseases and organ systems rather than patients, making care more patient or person centered would require no less than a paradigm shift in how we practice medicine^79^.

In some cases, current kidney care is inconsistent with patients’ preferences and values^23^. Consequently, dialysis is often associated with poor outcomes including low HRQoL. To improve patient-reported outcomes, incorporation of the patient’s needs and perspective into the medical care that is provided is essential^80^. Patient-centered care is adapted to facilitate integration of the patient’s lifestyle and community into the treatment plan. To be able to integrate both the patient’s and the clinician’s perspective, a model of culturally sensitive shared decision-making is encouraged^81^.

In practice, a person-centered care requires thoughtful, tailored kidney care that will often require balancing issues of survival and long-term health outcomes with maximizing HRQoL, symptom control, and physical and psychosocial function^82^. This approach essentially shifts the focus of shared-decision making away from guidelines and the evidence on which they are based toward what is important to each patient. In contrast to traditional care, physicians practicing patient-centered care may need to balance the management of symptoms (e.g., dizziness and fatigue) with optimal control of blood pressure (BP), anemia, and phosphate levels, with less emphasis being placed on maximizing long-term health outcomes, such as survival. As disease progresses, patients’ goals of care tend to shift to focus almost exclusively on HRQoL rather than survival, with a strong emphasis on emotional, social, and family support^80^^,^ 82.

In addition, to supporting a more individualized approach to decisions about dialysis initiation, some have also argued for greater flexibility in how we prescribe dialysis treatments for those receiving this therapy, which can of course shape patients’ upstream decisions about whether and when to start dialysis. For example, there are alternatives to standard thrice-weekly dialysis for patients who do not need or want the level of clearance that this would provide^83^. 

#### 8. Palliative dialysis

Palliative dialysis is a transition from a conventional disease-oriented focus on dialysis as rehabilitative treatment to an approach prioritizing comfort and alignment with patient preferences and goals of care to improve quality of life and reduce symptom burden for maintenance dialysis patients in their final year of life^84^. A palliative approach to dialysis delivery has been suggested for patients with limited life expectancy who wish to limit the burdens of treatment^84^^,^ 85. Palliative dialysis should be considered in specific clinical scenarios as i. patients on maintenance dialysis with limited life expectancy, ii. patient on maintenance dialysis who develops a severe illness that causes an abrupt decline in life expectancy, iii. patients that started on dialysis in the setting of acute kidney failure with an unclear life expectancy and goals of care, and iv. patient on maintenance dialysis with progressive functional and/or cognitive decline^83^.

This approach to palliative dialysis prioritizes HRQoL related to prevention and relief of symptoms and suffering rather than prolongation of life. Interventions are usually to control symptoms and distress and promoting wellbeing and social functioning. The requirement to sit for 4 hours doing hemodialysis can be almost intolerable for some patients and may contribute to functional and cognitive decline. Shorter dialysis with more frequent sessions may be more tolerable. Gentle intradialytic exercise, with or without the use of analgesics, can help manage symptoms such as restless legs and a sore back from inactivity, while helping to preserve function and improve mood^82^. In Table 6, there are some examples of approaches to common issues among maintenance dialysis patients in the current disease-focused dialysis delivery model.

**Table 6:** Comparison of approaches to common issues among the current disease-focused dialysis delivery model versus a palliative dialysis care model

Issue	Current Disease-Focused Metrics for Conventional Dialysis Care	A Patient-Centered and Palliative Approach to Dialysis Care
Vascular access	Creation and maintenance of an AV fistula	CVC is acceptable
Dialysis adequacy	Target small solute clearance based on current standards (Kt/V.1.2 for HD and Kt/V.1.7 for PD), intensifying the dialysis prescription as needed to achieve targets	Lower clearance acceptable if changes prescription increase demands inconsistent with patient preference. Taylor dialysis to minimize symptoms and treatment burden.
Cardiovascular disease	Treat CV risk factors, potentially targeting BP and dyslipidemia	Tolerate hypertension to avoid symptoms; limited use of medication to treat hypertension and dyslipidemia treatment
Mineral and bone disorder	Dietary counseling; binders to control hyperphosphatemia; vitamin D analogues with or without calcimimetics for secondary hyperparathyroidism	Limited restrictions; more permissive hyperphosphatemia and hyperparathyroidism
Nutrition	Encourage dietary protein intake while limiting potassium (if HD), sodium, and phosphorus intake	Dietary restrictions only to mitigate symptoms and improve quality of life.
Laboratory monitoring	Routine monthly laboratory tests	Minimal necessary
Drugs	Prescribed for treatment and prevention	Prescribed primarily to improve HRQoL or symptoms relief
Anemia management	IV iron and ESAs to achieve targets for Hb and TSAT/Ferritin	IV iron and ESAs only as needed
Symptom management	Only as needed	In a regular base

AV, arteriovenous; CVC, central venous catheter; HD, hemodialysis; PD, peritoneal dialysis. Modified from refs. 84 and 85.

As a patient-centered rather than disease oriented approach to the delivery of dialysis care among patients with limited life expectancy, a palliative approach to dialysis care could alleviate the suffering of such patients. Much work is needed to facilitate the incorporation of this approach into the existing dialysis delivery infrastructure in order to obtain its most effective use^83^.

#### 9. Incremental dialysis

Notwithstanding the potential benefits of an appropriate dialysis dose^86^, there is an increasing recognition that a significant burden of harm may arise from the delivery of conventional dialysis. While this is true across all patients on dialysis, the effects may be more pronounced in the frail elderly. These complications may accelerate any underlying cycle of frailty^87^. The commencement of hemodialysis (HD) is associated with increased levels of mortality, particularly in the elderly, along with loss of functional status in those who are most dependent^88^. This early period is associated with frequent episodes of hypotension even in units undertaking longer hours and using slower ultrafiltration rates^89^. Intradialytic episodes of hypotension appear to have deleterious effects on both cardiac^90^ and cerebral function^91^.

The concept of incremental HD is based on the simple idea of adjusting HD dose according to the metrics of residual kidney function (RKF). Indeed, most patients initiating dialysis have some degree of RKF, often a renal urea clearance (Kru) >3 mL/min and urine output (UO) >500 mL/day. It is a kind of dialysis that does the smooth “transition”, rather than abrupt “start”, from conservative management of CKD to dialysis therapy. It allows a reduced frequency of dialysis (one to twice a week)^92^.

Although literature on incremental HD is surprisingly small, it is growing quickly, especially in recent years. A pioneer study in Spain established a Kru limit of 2.5 mL/minute or more to initiate incremental HD^93^. This study showed that 35% of patients who started HD program twice a week had sufficient RFK to maintain this frequency of treatment^94^. The Kidney Diseases Outcomes Quality Initiative (KDOQI)^95^ suggests that minimum targets of adequacy of the dialysis dose (Kt/V) may be reduced in those with Kru ≥2 mL/min/1.73 m^2^.

Incremental HD has a lower burden of treatment and there appears to be no adverse clinical effects during the first years of dialysis^96^ in presence of a significant RKF. The advantages of incremental HD might be particularly important for elderly patients and others with short life expectancy, when the life experience or quality of life may be the priority for them.

#### 10. Forgoing dialysis

Withholding and withdrawal of dialysis is a very complex decision that should be made with the patient and involves clinicians’ skills and training to support this practice^97^. Furthermore, decisions about dialysis initiation or discontinuation must be considered under the light of bioethics principles by the nephrologist in the SDM process as follows.

- Autonomy - the patient, adequately informed of the risks and benefits of dialysis, should be able to decide whether or not dialysis will be made.
- No maleficence - it is our obligation not to harm our patients. Suffering is harm and we need to carefully assess whether dialysis will increase it.
- Beneficence - it is our duty to maximize benefits and minimize injury. To this end, we should select the patients most likely to benefit from dialysis, not only in terms of prolonging life, but also in maintaining the quality of life.
- Justice - we are obliged to offer our patients equal opportunities and allocation of available resources^98^.

Some guidelines support clinicians, patients, and families with evidence about the benefits and burdens of dialysis, bring recommendations for quality decision-making about treatments, and establish strategies to help clinicians implement the guideline recommendations^3^^,^ 4.

It is up to the nephrologist and interdisciplinary team that care for the patient to look for potentially correctable factors that can contribute to the decision to forgo treatments, such as depression, other distressing symptoms such as pain, and potentially reversible social factors. Table 7 shows the recommended situations for dialysis withdrawal^5^.

**Table 7:** Recommendations for withdrawal of dialysis

1. Patients with decisional capacity, who are fully informed and make voluntary choices, refuse dialysis or request dialysis to be discontinued.
2. Patients who have no more decision-making ability and who have previously expressed refusal to dialysis through appropriate ACP.
3. Patients who are no longer able to make decisions and whose legal representatives refuse dialysis or request that they be discontinued.
4. Patients with irreversible and profound neurological impairment, so that they do not show signs of thought, sensation, intentional behavior, and self-awareness and the environment.
5. Patients with clinical and functional deterioration, with evidence of intolerability to the dialysis procedure (maleficence).

ACP, advance care planning (adapted from reference 5).

Ensuring access to appropriate palliative care is an integral part of clinical assistance after the decision to withdraw dialysis^5^.

#### 11. End-of-life (EOL) care

Refers to the care given to patients in the last days or weeks of life when clinical deterioration is likely to be irreversible and death imminent. It includes physical, spiritual, and psychosocial assessment, and care and treatment provided by an interdisciplinary team with knowledge and skills in this area. This also aligns support for family members / caregivers and care of the patient’s body after death and grieving of relatives / caregivers.

Table 8 presents the most common symptoms in the final phase of life and therapeutic approach.

**Table 8:** Symptoms and therapeutic measures in the last days of life

Symptom	Intervention
Nausea and vomiting	Haloperidol SC 0,5 to 1.0 mg 8 hourly
	Levomepromazine SC 2.5 to 5 mg hourly
Respiratory secretions	Hyoscine butilbromide SC 20 mg, hourly as required (up to 120 mg in 24h)
Anxiety and distress	Midazolam SC 2 mg as required hourly
	Lorazepam sublingual 0.5 mg 8 hourly as required
Dyspnea	Fentanyl 25–50 µg subcutaneous 2 hourly as required (first choice)
	Morphine 1.5–2.5 mg sub subcutaneous cut 2 hourly as required
	Diuretic (if applicable), ventilator (in face), and relaxation techniques
Delirium	Haloperidol 0.5 mg to 2 mg 8 hourly
Terminal agitation	Midazolam SC 10 to 20 mg over 24 h plus midazolam SC 5 mg hourly, as required

Adapted from reference 99.

Some algorithms were developed elsewhere specifically for CKD patients at the end-of-life to address symptoms like breathlessness, pain, nausea and vomiting, respiratory secretions, and agitation and restlessness that can be freely accessed online^99^

## Conclusion

Over the past 20 years there were a great advance in renal palliative care that came with a better understanding of the basic pathophysiology and management of symptoms in CKD, prognostication tools, and improvement in difficult communication. Besides, with the demographic change all around the world, there is a growing number of patients opting for conservative care without dialysis by their own option or medical recommendation. In addition, dialysis is changing from a disease-centered to person-centered treatment, where a health literate patient choose how, when, and where they desire to do it. Foregoing dialysis seems to be increasing despite dialysis discontinuation still being a conundrum to most nephrologists. Despite the development of palliative care, there is an enormous gap between theory and practice in nephrology, and the integration of a palliative care service to the usual renal care is still incipient or non-existent in Brazil. Therefore, one could argue that it is mandatory that scientific societies and governments be involved in creating policies for a sustainable health system by means of education and training in renal palliative care.
